# Correction: A universal chemical potential for sulfur vapours

**DOI:** 10.1039/c6sc90058e

**Published:** 2016-08-26

**Authors:** Adam J. Jackson, Davide Tiana, Aron Walsh

**Affiliations:** a Centre for Sustainable Chemical Technologies, Dept. of Chemistry, University of Bath Claverton Down Bath BA2 7AY UK a.walsh@bath.ac.uk; b Global E3 Institute, Department of Materials Science and Engineering, Yonsei University Seoul 120-749 Korea

## Abstract

Correction for ‘A universal chemical potential for sulfur vapours’ by Adam J. Jackson *et al.*, *Chem. Sci.*, 2016, **7**, 1082–1092.

The authors regret that the *Parameterisation* section of the original article requires correction. On page 1090 of the original article, two indices within the in-line expression on lines 6–7 are incorrect. The amended version of this equation is as follows:

*T*_tr_ = 5.077 × 10^2^ + 7.272 × 10^1^ log_10_ *P* − 8.295(log_10_ *P*)^2^ + 1.828(log_10_ *P*)^3^.

Additionally, the authors wish to draw readers' attention to an inconsistency in the units for the energy terms used in this section, with the following Gaussian correction on lines 8–9 provided in units of ‘J mol^−1^’ and the other energy terms provided in units of ‘eV per atom’.

*a*(*P*) = 1.414 × 10^3^ − 2.041 × 10^2^ log_10_ *P* + 6.663 × 10^1^(log_10_ *P*)^2^

The supplementary information for the original article has been updated with a clarification of the parameterisation, including values for the energy terms in units of both ‘J mol^−1^’ and ‘eV per atom’. The authors thank Volodymyr Kosyak and Jonathan Scragg for highlighting these issues.

The Royal Society of Chemistry apologises for these errors and any consequent inconvenience to authors and readers.

## Supplementary Material

